# Area-Wide Suppression of the Mediterranean Fruit Fly, *Ceratitis capitata,* and the Oriental Fruit Fly, *Bactrocera dorsalis*, in Kamuela, Hawaii

**DOI:** 10.1673/031.010.13501

**Published:** 2010-08-17

**Authors:** Roger I. Vargas, Jaime C. Piñero, Ronald F. L. Mau, Eric B. Jang, Lester M. Klungness, Donald O. McInnis, Ernest B. Harris, Grant T. McQuate, Renato C. Bautista, Lyle Wong

**Affiliations:** ^1^ U.S. Pacific Basin Agricultural Research Center, USDA-ARS, P.O. Box 4459, Hilo, Hawaii, 96720, USA; ^2^ University of Hawaii at Manoa, College of Trop. Agric. & Human Resources. 3050 Maile Way, Honolulu, Hawaii, 96822, USA; ^3^ U.S. Pacific Basin Agricultural Research Center, USDA-ARS, 2727 Woodlawn Drive, Honolulu, Hawaii, 96822, USA; ^4^ Plant Pest Control Branch, Plant Industry Division, Hawaii Department of Agriculture, 1428 South King Street, Honolulu, Hawaii, 96814, USA; ^5^ Lincoln University of Missouri, Cooperative Research and Extension, Allen Hall 212, Jefferson City, Missouri, 65101, USA

**Keywords:** bait spray, Integrated Pest Management, male annihilation, monitoring, Tephritidae

## Abstract

The United States Department of Agriculture's Agricultural Research Service initiated an area-wide fruit fly management program in Hawaii in 2000. The first demonstration site was established in Kamuela, Hawaii, USA. This paper documents suppression of the Mediterranean fruit fly, *Ceratitis capitata* (Wiedemann), and the oriental fruit fly, *Bactrocera dorsalis* (Hendel) (Diptera: Tephritidae), in a 40 km^2^ area containing urban, rural and agricultural zones during a 6 year period. The suppression techniques included sanitation, GF-120 NF Naturalyte Fruit Fly Bait sprays, male annihilation, Biolure® traps, and parasitoids against *C. capitata* and *B. dorsalis.* In addition, small numbers of sterile males were released against *B. dorsalis.* Substantial reductions in fruit infestation levels were achieved for both species (90.7 and 60.7% for *C. capitata* and *B. dorsalis,* respectively) throughout the treatment period. Fruit fly captures in the 40 km^2^ treatment area were significantly lower during the 6 year period than those recorded in three non-treated areas. The strategy of combining suppression techniques in an area-wide approach is discussed.

## Introduction

An area-wide insect control program is a long-term campaign against an insect pest population throughout its entire range with the objective of reducing the insect population to a non-economic status (Lindquist 2000). The importance of area-wide integrated pest management for suppression and/or eradication of tephritid flies has been documented by Koyama et al. (2004), Dhillon et al. (2005), Mau et al. (2007), Vargas et al. (2007, 2008), and Jang et al. (2008).

The use of single suppression techniques to reduce or eradicate fruit flies from an area where they are well established has proven insufficient in many cases, and consequently, most successful programs have resorted to the use of multiple suppression techniques. For example, in 1994, the government of Taiwan launched a nation-wide program to eradicate the oriental fruit fly, *Bactrocera dorsalis* (Hendel) (Diptera: Tephritidae), from the island. By the year 2002, they applied 42 metric tons of methyl eugenol and accomplished 75% suppression island-wide, but they were not able to achieve further reductions with male annihilation alone (E. Chang, personal communication). They subsequently incorporated bait sprays, sanitation, and fruit bagging to concentrate their efforts in an area-wide multi-technique approach and accomplished further suppression of the *B. dorsalis* population (Huang 2007). A second example of a successful eradication program that relied on an integrated approach was the island country of Mauritius, following an accidental introduction of *B. dorsalis* in 1996. With the support of the International Atomic Energy Agency, Mauritius undertook an eradication program that incorporated bait sprays, methyl eugenol, and fruit disposal. The result of this program was the total elimination of *B. dorsalis* by 1999 (Seewooruthun et al. 2000).

In 2000, the Hawaii Fruit Fly Area-Wide Pest Management program was implemented by the United States Department of Agriculture Agriculture Research Service (USDA-ARS) to develop and integrate sustainable fruit fly management methods with area-wide demonstration projects. An important goal of this program was to transfer economical and ecologically sound technologies to the growers (Mau et al. 2007). This program began with an effort to identify areas where fruit flies most impacted agriculture, as well as areas where growers would be most cooperative and supportive of the program, such that suppression would be successful. To that end, a survey was initiated in 1999 on five islands of Hawaii. The initial site selection, as well as the results concerning suppression of the first species targeted, which was the melon fly, *Bactrocera Cucurbitae* (Coquillett), are described in Jang et al. (2008). The implementation of the area-wide program on other Hawaiian islands is reported by Mau et al. (2003a, 2003b, 2007) and Vargas et al. (2007, 2008). Here, the impact of the techniques used to suppress both *Ceratitis capitata* (Wiedemann) (Diptera: Tephritidae) and *B. dorsalis* in Kamuela, Hawaii, the first target area selected for program implementation, is described.

## Materials and Methods

### Target area selection

Based on the results of surveys throughout the state of Hawaii, Kamuela was chosen as the first target area on Hawaii Island. Selection of this site was based on the more manageable fruit fly populations and a grower-based community that actively supported the program. Two additional sites (Kunia, Oahu and Kula, Maui) were selected on other islands, but this report summarizes results for the Kamuela site.

### Baseline data

A trapping survey was conducted in nine sites in Lalamilo Farm Lots in Kamuela to determine the baseline population of the two target species. For each trapping site, there were five traps baited with five different attractants, deployed between 3 and 6 m of each other. These traps were monitored on a biweekly basis for 6 months to 1 yr before suppression began, and monitoring continued throughout the suppression program.

### Target species selection

The first species targeted in this program was *B. Cucurbitae,* and the results for that species are presented in Jang et al. (2008). The second species selected was *C. capitata,* based on its moderate population level that peaks in summer due to the presence of backyard plantings of *Prunus* spp. (peach, plum, etc.) and *Diospyros kaki* L. (persimmon), most of which were for home consumption, although some fruits were marketed commercially. *B. dorsalis* was the third species targeted.

### Suppression technologies

Five suppression technologies (sanitation, bait spraying, male annihilation, and sterile male and parasitoid releases) were utilized in this program. In general terms, the areas with the highest number of fly captures received the most applications of suppression treatments.

**1) Sanitation** was achieved by using augmentoria (Klungness et al. 2005; Jang et al. 2007) and/or disposal of culled fruit by the growers using bags that were removed from the farm. Fifteen farms were initially included but level of grower cooperation varied from farm to farm (reported in Jang et al. 2007). No attempts were made to apply sanitation to wild hosts.

**2) Bait spraying** was initially accomplished with GF-120 Fruit Fly Bait (Dow AgroSciences, LLC, www.dowagro.com), and later with the organic formulation GF-120 NF Naturalyte® NF Fruit Fly Bait certified by the Organic Materials Review Institute (www.omri.org). The effectiveness of this reduced-risk insecticidal bait against tephritid flies in Hawaii has been recently demonstrated by Peck and McQuate (2000), Vargas et al. (2001), McQuate et al. (2005a, 2005b), Prokopy et al. (2003, 2004), Jang et al. (2008), and Piñero et al. (2009, 2010). The weekly bait sprays were initiated on 27 July 2001 and were interrupted on 17 November 2004. Then they were resumed on 6 May 2005 and continued weekly until 7 July 2005. This bait was applied at a rate of between 800 ml to 56.5 liters per week, to either host plants of *B. dorsalis* and *C. capitata* or to vegetation near host plants. Some farmers maintained a variable number of MultiLure® traps (Better World Manufacturing) baited with Biolure® (Suterra LLC, www.suterra.com), a 3-component fruit fly food lure, for trapping male and female *C. capitata.*

**3) Male annihilation** was accomplished by deploying traps baited with the male-specific lures trimedlure (1, 1-dimethylethyl 4 (or 5)chloro-2-methylcyclohexanecarboxylate) against *C. capitata* and methyl eugenol (1, 2-dimethoxy-4-(2-propenyl)benzene) against *B. dorsalis*. Lures were deployed in plastic matrices of 2 and 4 g (a.i.) for methyl eugenol and trimedlure, respectively (Scentry Biologicals, www.scentry.com) using plastic buckets (Highland Plastics, www.highlandplasticsinc.com). Bucket traps are fully described in Vargas et al. (2003), but in short, they were 5 liters in capacity for *B. dorsalis* and 1 liter in capacity for *C. capitata.* Each trap had four 1.9 cm entrance holes on the side and four 0.3 cm drain holes on the bottom. The toxicant used was 2,2-Dichlorovinyl dimethyl phosphate (DDVP) (Vaportape® II, Hercon Environmental, www.herconenviron.com). Each baseline survey site contained one trap for each species.

**Figure 1.**
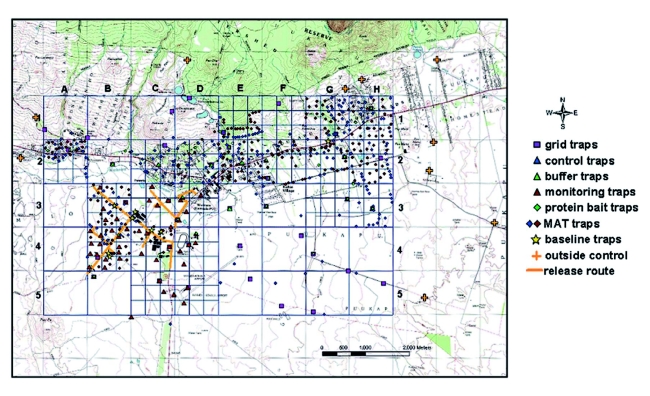
Topographic map of target area grid in Kamuela, Hawaii Island, showing all trap types deployed. Traps depicted include protein bait and the male-specific lure methyl eugenol (against *B. dorsalis*) traps deployed as the baseline, as well as grid and male annihilation treatment traps. Three additional trap types (control, buffer, and treatment areas) were deployed to monitor the sterile fly release. Some control traps placed outside the grid (= outside control) are also indicated. High quality figures are available online.

**4) Release of sterile *B. dorsalis* males** that were produced by the USDA-ARS, US Pacific Basin Agricultural Research Center Fruit Fly Center, Honolulu, Hawaii (McInnis et al. 2004, 2006, 2007). Sterile males were shipped to Hawaii Island between 29 January and 21 August 2005. The actual releases occurred on a weekly basis between 2 February and 29 September 2005. The number of flies released varied between 99,600 and 595,800 per week with an estimated total of 11,556,000 flies released.

**5) Parasitoid** augmentative releases were conducted using *Fopius arisanus* (Sonan) (Hymenoptera: Braconidae) produced at the USDA Manoa lab (Bautista et al. 1999). Weekly releases took place between 26 March 2003 and 10 January 2004 with numbers varying from 4,736 to 182,344 wasps/week.

### Area grid monitoring

The control program began with the establishment of a 40 km^2^ grid, including the Lalamilo Farm Lots and a range of other land-use categories. Grid divisions were named Al to A5 through Hl to H5 (Figure 1). Initially the grid was plotted on a map and male lure monitoring traps were deployed at a density of 1 set of traps (i.e., trimedlure and methyl eugenol) per km^2^. Permission to enter private property to service the traps was obtained from individual owners. These ‘grid traps’ baited with male lures became the standard of comparison over time for subsequent deployment of suppression techniques, providing data for the 6 years of the project. For safety reasons (WW II unexploded ordinance was found at the end of 2000), grid survey traps covering 3 km^2^ had to be removed from quadrant A3, A4, and A5. When suppression of *B. dorsalis* was well underway, it was determined that 8 additional traps per km^2^ needed to be added on the northeastern side of the 40 km^2^ grid in order to detect migrating flies entering the grid area. Traps were monitored on a biweekly basis. Lures were replaced every 3 months (Vargas et al. 2005).

### Geographic Information System

Soon after the deployment of the initial grid traps, a geographic information systems (GIS) approach was adopted in order to support the trapping program. This included establishing Geographic Positioning System (GPS) coordinates for each grid trap, as well as for main host plants throughout the grid area. Garmin GPS 12 units (Garmin International, Inc., www.garmin.com) were used to record GPS coordinates. Later, the coordinates were transferred to ARCInfo® (Environmental Systems Research Institute, www.esri.com) mapping software. Data were keypunched directly into ARCInfo® datafiles or transcribed to Microsoft Excel® spreadsheets and imported to ARCInfo® for mapping. Graphical presentations were done with Sigma Plot® (SPSS Inc., www.spss.com) and Microsoft Excel®.

### Protein bait monitoring traps

For each site, one yellow dome trap (Better World Manufacturing) containing either Mesoferm® (Corn Products International Inc., www.cornproducts.com) or NuLure® (Miller Chemical & Fertilizer Corp., www.millerchemical.com) was deployed to monitor female populations. Because food-baited traps are known to attract fruit flies from relatively short distances, these monitoring traps were expected to represent a good estimate of populations present in the vicinity. In addition to the protein bait traps deployed within the grid, the staff deployed protein bait traps at a density of ≥ 2 per actively-fruiting crop site including wild, garden or commercial host plants. These additional traps were baited with a new bait product, Solulys (Roquette America Inc., www.roquette.com) buffered with 5% borax (U.S. Borax, Inc., www.borax.com). This bait was mixed with up to 30% polypropylene glycol to prevent desiccation without impacting trap captures. Traps were serviced weekly or biweekly depending on the availability of staff.

### Plant host mapping and fruit sampling

The host mapping served three purposes: (1) collecting fruit for rearing of fruit flies, (2) documenting the fruiting phenology throughout the grid, and (3) locating and mapping all potential host plants. Numbers of fruits collected from gardens, orchards, and commercial crops varied from 10 to 90 per site at ca. 1–2 week intervals. Frequency varied depending on the work load and availability of staff. Table 1 presents the species of fruit, the sum of the sites, and the number of fruit collected over the sampling dates, as well as the number of flies of each species recovered.

For the first 3 years, fruit sampling was restricted to damaged fruit. The rationale for this was to maximize chances of finding infested fruit within logistical constraints. In addition, for 1 year in the middle of the suppression program (28 August 2002 – 27 August 2003), each observer recorded how many fruit were inspected before damaged fruit was found, and infested fruits were taken to the lab to rear larvae. This process, often called presence-absence sampling, was repeated one or more times at each sampling site. In the absence of damaged fruit at a site, the number of inspected fruits was recorded.

This presence-absence sampling method provided three measurements: (1) percentage of all fruit samples that were infested per date (hereafter called “infested% of sample”), (2) percentage of the visibly damaged fruit that actually contained larvae (specifically, percent of damaged fruits collected that were infested, hereafter called “infested% of damaged fruit”), and (3) percentage of all fruits observed that actually had larvae in the damaged fruit (hereafter called “infested% of observed fruit”). In their search for fruit, the crew discovered new host plant loci, and these in turn yielded new sources of fruit. Thus the database grew to allow calculation of host acreage.

When the project's primary emphasis transitioned from *B. Cucurbitae* to *B. dorsalis* in 2003, the fruit collections changed to fully randomized 1 m^2^ sampling at sites randomly selected from the grid. Twenty-two km^2^ of the 44 km^2^ in the extended grid were determined to be areas where there were host plants for *B. dorsalis.* These 22 km^2^ were further subdivided into 9 sub-quadrants. The sub-quadrants from which fruit was to be collected were selected from a random numbers table. This sampling method continued between 22 July 2003 and 1 November 2005. However, this method proved to be inadequate to accurately sample such a diversity of clustered plant hosts over such a large area. Therefore, in order to increase collection of infested host fruit, the sampling scheme returned to the aforementioned methods that included the presence-absence sampling method.

**Table 1.**
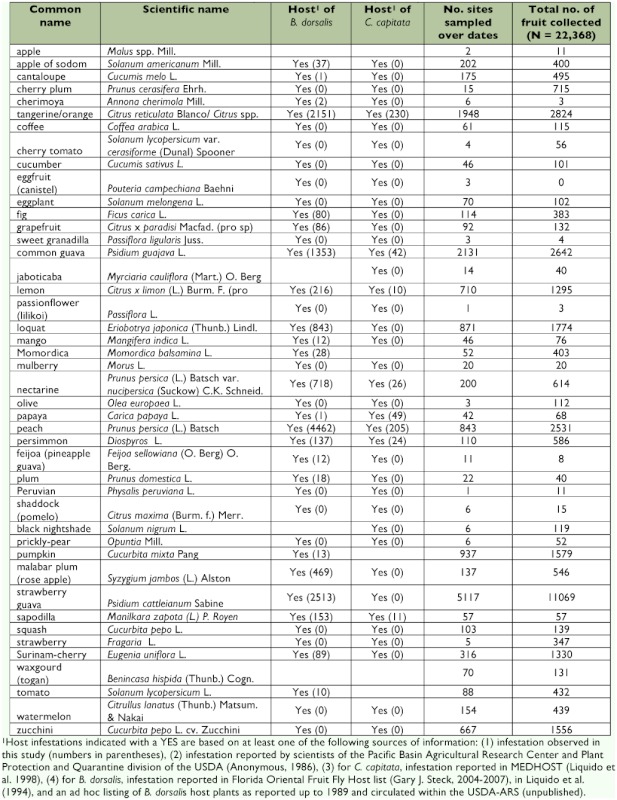
Host fruit collected over the course of the suppression program for rearing out fruit fly larvae. For each fly species, the total number of infested fruit is shown in parentheses. Scientific names of plants are from the PLANT Database (USDA, Natural Resources Conservation Service). Plant common names shown in parentheses indicate local (Hawaiian) names.

### Technology transfer

A primary objective of the Hawaii Fruit Fly Area-Wide Pest Management program was to transfer new safer technologies rapidly to growers. Therefore, throughout the suppression period, commercial growers were encouraged to participate in the control measures by applying bait sprays, practicing sanitation, tilling quickly after harvest, and deploying their own male annihilation traps. To that end, weekly updates of the fly populations in their fields were provided. Growers were also supplied with protein bait (GF-120 Fruit Fly Bait and later GF-120 NF Naturalyte Fruit Fly Bait) (max. 298.4 liters per grower) and with augmentoria, and they were also given general advice. In areas where the growers could not apply the techniques themselves, USDA personnel carried out all the above techniques except sanitation (Table 2). Even though ca. 20% of the 40 km^2^ grid area was zoned agricultural land, only 1.5% contained active farms (of which only 0.44% contained fruit fly hosts). The remaining residential rural and forest land contained host plants for all species of tephritid flies currently present in the Hawaiian Islands (Vargas et al. 2008).

### Assessment

The combined impact of sanitation, bait spraying, male annihilation, SIT and parasitoid releases was determined first by examination of the male lure and protein bait trap catch on a bi-weekly basis, as well as by fruit infestation. In addition, to provide a quantitative measure of the impact of the suppression program, three sites (Lakeland (912 MASL), Waikoloa (420 MASL), and Kawaihae (10 MASL)) were selected outside the 40 km^2^ target area in Kamuela (900 recorded over a 6-year period. Between the mean of the first and last 10 observation dates, there was a 90.7% reduction in infestation.

**Table 2.**
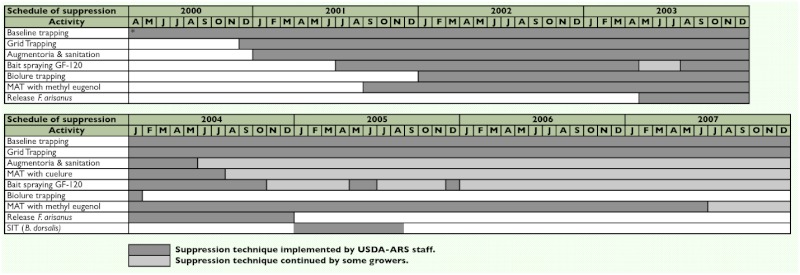
Implementation schedule of area-wide fruit fly suppression in Kamuela, Hawaii

**Figure 2.**
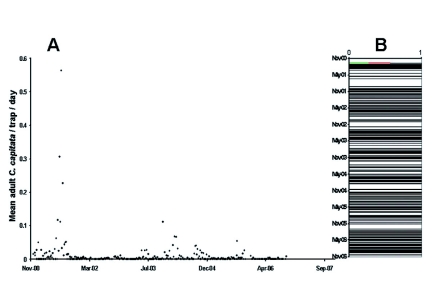
(A) Mean captures (flies/trap/day) of adult *C. capitata* in grid traps baited with the male-specific lure trimedlure according to trapping date. (B) Frequency of zero captures (black horizontal lines), maximum f/t/d value (red line) and predicted maximum f/t/d value (green line). High quality figures are available online.

**Figure 3.**
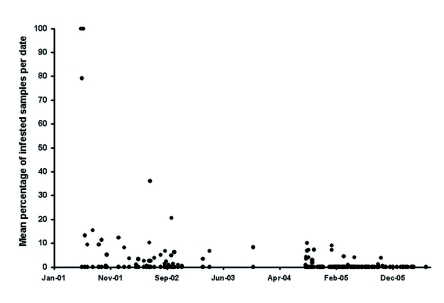
Percent infestation of fruit by *C. capitata* over a 5 year period. High quality figures are available online.

The presence-absence sampling of *C. capitata* hosts did not begin until 7 September 2004 (infested % of observed fruit). The latter is the best estimate of the actual percent infestation of all fruit and indicates a very low level of infestation (highest value was 1.89%) even after cessation of trapping with Biolure® and bait sprays.

**Figure 4.**
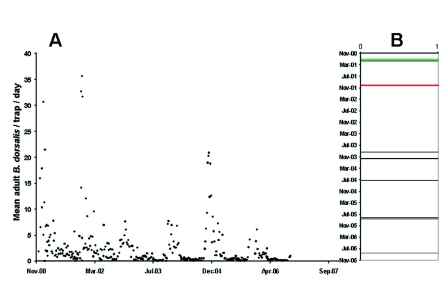
(A) Mean captures of adult *B. dorsalis* in grid traps baited with the male-specific lure methyl eugenol according to trapping date. (B) Frequency of zero captures. High quality figures are available online.

**Figure 5.**
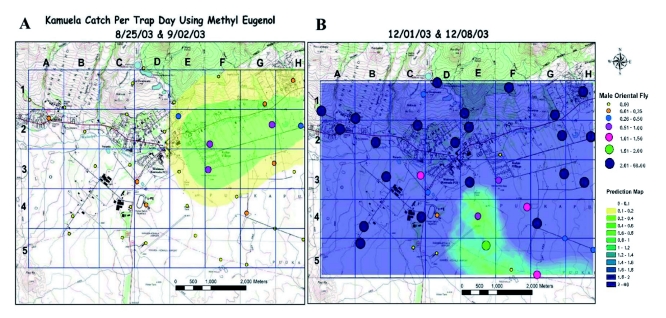
Relative captures of adult *B. dorsalis* in grid traps during (A) late August and early September 2003, and (B) early December 2003 in Kamuela, Hawaii Island. High quality figures are available online.

***Bactrocera dorsalis*.** Figure 4 summarizes the combined effect of the suppression treatments on the *B. dorsalis* population as determined by trap captures. Figure 4B illustrates that the mean number of male captures over the entire target area very seldom reached zero. Incursions of *B. dorsalis* began in the eastern portion of the grid (Figure 5A), and by December, the population typically became saturated throughout the areas where there were host plants (Figure 5B). These images clearly illustrate the gradual movement of the flies into the higher elevation (> 900 MASL) areas as the late season wild host fruit ripened. In spite of the cyclic migrations of *B. dorsalis* into Kamuela, the suppression efforts were able to reduce the peak November capture rate of 35.6 f/t/d to a mean of 0.15 ± 0.03 f/t/d between 5 June and 28 August 2006 (a reduction of 99.5%). This was after the time when maximum bait spray and male annihilation treatments occurred, and after release of *F. arisanus* and sterile *B. dorsalis* males. More realistically, averaging the mean capture rate before (3.30 ± 0.44) and after mid-project (3 October 2003) (1.82 ± 0.27), the difference is a 44.9% reduction in *B. dorsalis* captures per trap per day over the 6 years.

**Figure 6.**
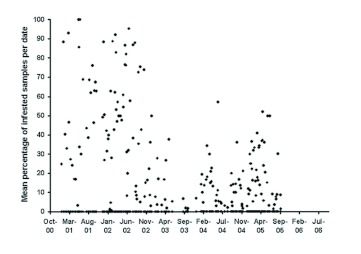
Percent infestation of fruit by *B. dorsalis* over a 5 year period. High quality figures are available online.

In terms of fruit infestation, a total of 13,679 *B. dorsalis* were recovered from the 29,811 fruit that were collected. Figure 6 presents the actual mean percent infestation values recorded over a 6-year period. The mean percent infestation by *B. dorsalis* from the beginning of the project to the mid-point of bait spray application was 42.18 ± 2.92%. From the mid-point to the end of bait spray application the mean infestation% was reduced to 16.59 ± 1.43%. That is a reduction of 60.67%.

For the comparison of data collected in three sites (Lakeland, Waikoloa, and Kawaihae) located outside the 40 km^2^ target area versus data from Kamuela, Table 3 reveals that the populations of *C. capitata* and *B. dorsalis* were significantly suppressed in Kamuela compared to the other three untreated sites over the 6 years, regardless of elevation, since Lakeland fruit fly captures differed from those in Kamuela where elevations were similar. Overall, captures of *C. capitata* and *B. dorsalis* in Kamuela were 97.5% and 81.2% lower, respectively, when compared to the three control sites combined.

## Discussion

The USDA-ARS has been a major developer of fruit fly control techniques for use in the continental United States and around the world. Much of this work, specifically against *C. capitata* and *B. dorsalis*, has been conducted in Hawaii, but until this program, no one had packaged the techniques and adapted them for use in Hawaii. Rather than eradication, the Hawaii Area-Wide Pest Management project was planned as an area-wide integrated pest management (IPM) program. One of the principal differences between IPM and eradication is that IPM sets the goal of keeping pest damage below an economically significant threshold rather than trying to eliminate every last fly.

Results of the 6-year Area-Wide Pest Management program in Kamuela suggest that the multiple-technique approach effectively reduced *C. capitata* and *B. dorsalis* populations throughout the entire area. The process began with a strategic deployment of monitoring traps and host-plant data collection in order to identify the areas of highest fruit fly activity. Data were then used to target the deployment of suppression techniques in areas of highest fruit fly numbers. Suppression techniques included sanitation, GF-120 NF Naturalyte Fruit Fly Bait sprays, male annihilation traps, Biolure® traps, and parasitoids against *C. capitata* and *B. dorsalis.* In addition, relatively small numbers of sterile males were released against *B. dorsalis.*

Overall, substantial reductions in fruit infestation levels were achieved for both species (90.7 and 60.7% for *C. capitata* and *B. dorsalis,* respectively). Fruit fly captures in the 40 km^2^ treatment area were significantly lower during the 6 year period than those recorded in three non-treated areas, an excellent indication of the efficacy of the suppression program.

During the initial phases of the program, growers were provided with IPM materials, supplies, and advice needed to manage the fruit fly pests. Eventually, they graduated to obtaining their own supplies, and the program is continuing under their own initiative (Mau et al. 2007). Although the farmers and home gardeners in Kamuela actively participated in the program, the USDA-ARS staff carried out much of the GF-120 NF Naturalyte Fruit Fly Bait and male annihilation treatments throughout the project because of the large areas of wild hosts such as strawberry guava, one of the dominant host plant species of *B. dorsalis* in the Island. The Kamuela program was a landmark demonstration project for the state of Hawaii. A large measure of the success of the program rests with this initial group of cooperators. Not only did they prove the viability of the area-wide concept, but they served as secondary information distributors, generating a chain reaction of interest and enrollment in the program by themselves (Mau et al. 2007; Vargas et al. 2008).

**Table 3.**
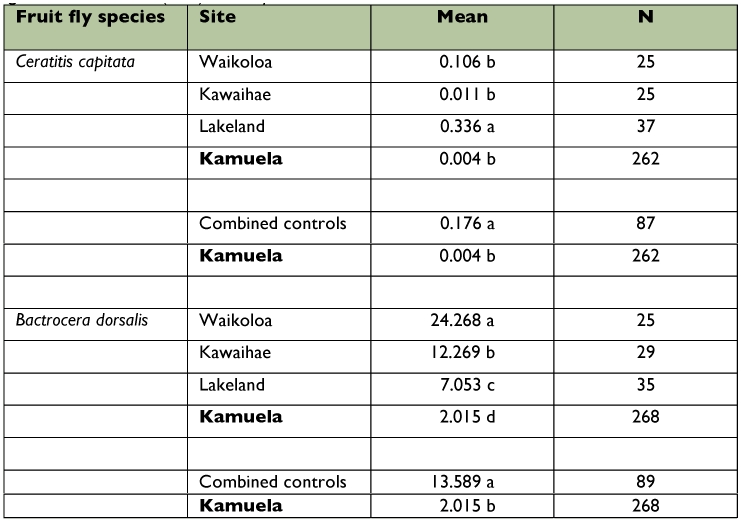
Comparison of treated area (Kamuela, Hawaii Island) to three control areas. Data are provided in mean no. males per trap per day (2001–2006). For each fly species, values with the same letter are not significantly different according to Fisher's Least Significant Difference (LSD) test at *p* = 0.05.

In action programs of this type where multiple tactics are used it is often hard to quantify the impact of individual components. However, the impact of individual components on fruit fly suppression was documented in separate controlled tests in Hawaii. For example, the importance of sanitation was quantified by Klungness et al. (2005) and more recently in two relatively large-scale studies by Piñero et al. (2009, 2010). The effects of protein bait sprays using GF-120 NF Naturalyte Fruit Fly Bait against *C. capitata* were reported by Peck and McQuate (2000a) and also against *B. dorsalis* by Piñero et al. (2009, 2010). Likewise, the effectiveness of Biolure® traps against *C. capitata* was documented by McQuate et al. (2005a), and the effectiveness of male annihilation traps was reported by Vargas et al. (2003). The impact of sterile fly and parasitoid releases on infestation by *B. dorsalis* was difficult to determine in the Kamuela program because of the small numbers of parasitoids and sterile flies released and the short release periods. Nonetheless, the effectiveness of small releases of *F. arisanus* fly releases against *B. dorsalis* was documented by Vargas et al. (2007), and the effectiveness of small numbers of sterile fly releases against *B. Cucurbitae* were documented by McInnis et al. (2007) and Jang et al. (2008)

In summary, the effectiveness of combining suppression techniques in an area-wide approach against *C. capitata* and *B. dorsalis* was demonstrated in the Kamuela area of Hawaii Island during a 6 year period. The Hawaii Fruit Fly Area-Wide Pest Management program has made major economic contributions to agriculture in Hawaii, and promoted production of a greater diversity of crops. In addition, by allowing farmers to make significant cuts in pesticide use, the program is helping improve Hawaii's environment and sustain open space, which contributes to maintaining the islands' tourism (McGregor 2007).
